# Supplementary material for: Ferromagnetism and giant magnetoresistance in zinc-blende FeAs monolayers embedded in semiconductor structures
Source: Nat Commun. 2021 Jul 7;12:4201. doi: 10.1038/s41467-021-24190-w (PMC8263727; doi:10.1038/s41467-021-24190-w)
Supplement: Supplementary file 1 — Supplementary Information [file 41467_2021_24190_MOESM1_ESM.pdf]

Supplementary Information

Ferromagnetism and giant magnetoresistance in zinc-blende FeAs monolayers embedded in semiconductor structures

Le Duc Anh[1,2,3*], Taiki Hayakawa[1], Yuji Nakagawa[4], Hikari Shinya[5,6,7], Tetsuya Fukushima[7,8,9], Masaki Kobayashi[1,9], Hiroshi Katayama-Yoshida[9], Yoshihiro Iwasa[4,10], and Masaaki Tanaka[1,9,*]

[1]*Dept. of Electrical Engineering and Information Systems, The University of Tokyo, Japan*
[2]*Institute of Engineering Innovation, The University of Tokyo, Japan*
[3]*PRESTO, Japan Science and Technology Agency, Japan*
[4]*QPEC & Dept. of Applied Physics, The University of Tokyo, Japan*
[5]*Research Institute of Electrical Communication, Tohoku University, Japan*
[6]*Center for Spintronics Research Network (CSRN), Tohoku University, Japan*
[7]*Center for Spintronics Research Network (CSRN), Osaka University, Japan*
[8]*Institute for Solid State Physics, The University of Tokyo, Japan*
[9]*Center for Spintronics Research Network (CSRN), The University of Tokyo, Japan*
[10]*RIKEN Center for Emergent Matter Science (CEMS), Japan*

Supplementary Figures and Table

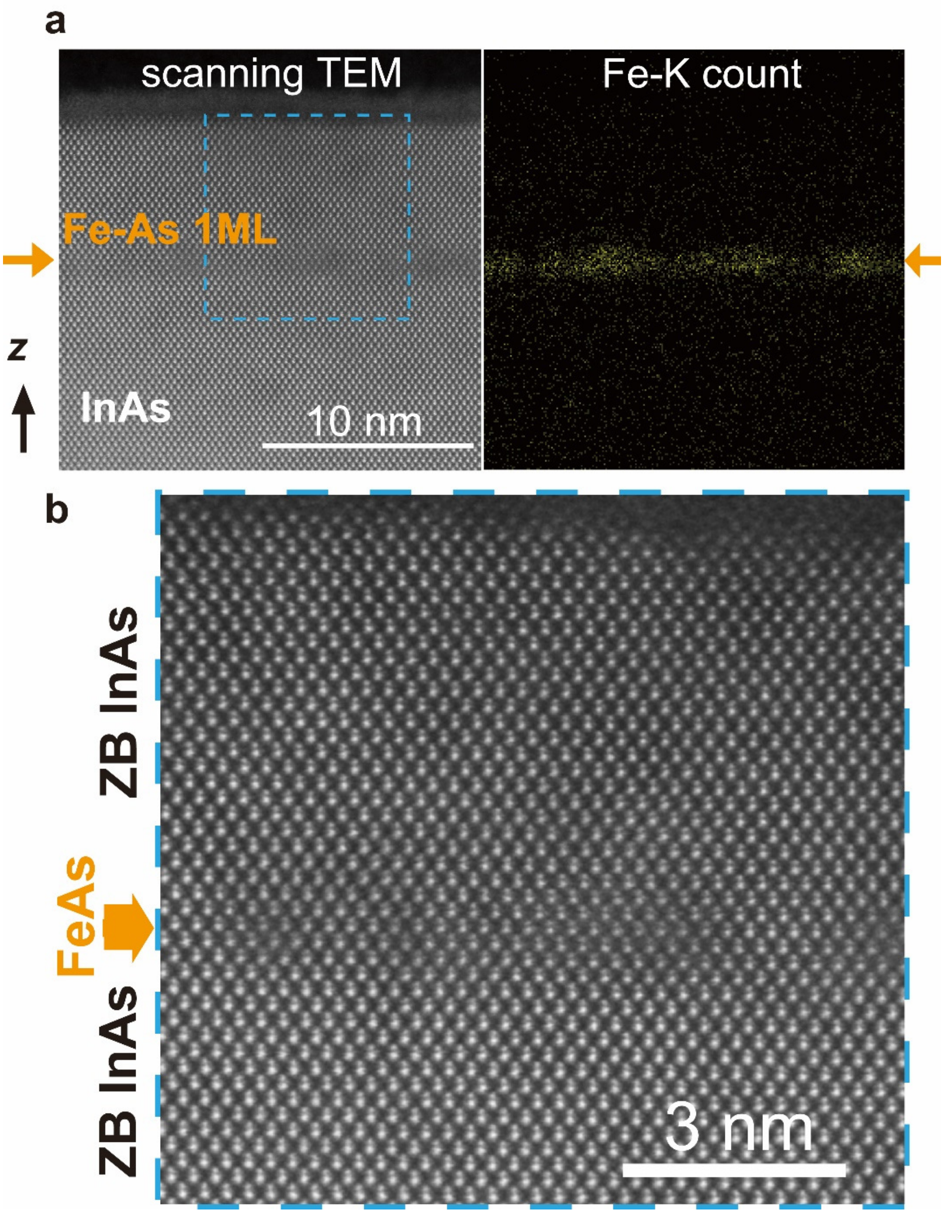

Supplementary Fig S1| (a) Scanning transmission electron microscopy (STEM) lattice image (left panel) and energy dispersive X-ray spectroscopy (EDX) mapping of Fe atoms (yellow points, right panel) in the sample of one FeAs layer embedded in an InAs matrix. From the EDX mapping, the FeAs layer position can be identified. (b) Magnified STEM lattice image of the FeAs layer embedded in the InAs matrix. The whole area, including the FeAs layer, preserves the zinc-blende crystal structure. It is noteworthy that in the in-plane [-110] direction, the FeAs layer seems to periodically broaden along the growth direction (*z* axis) with a period of ~3 nm. This possibly results from spinodal decomposition of Fe.

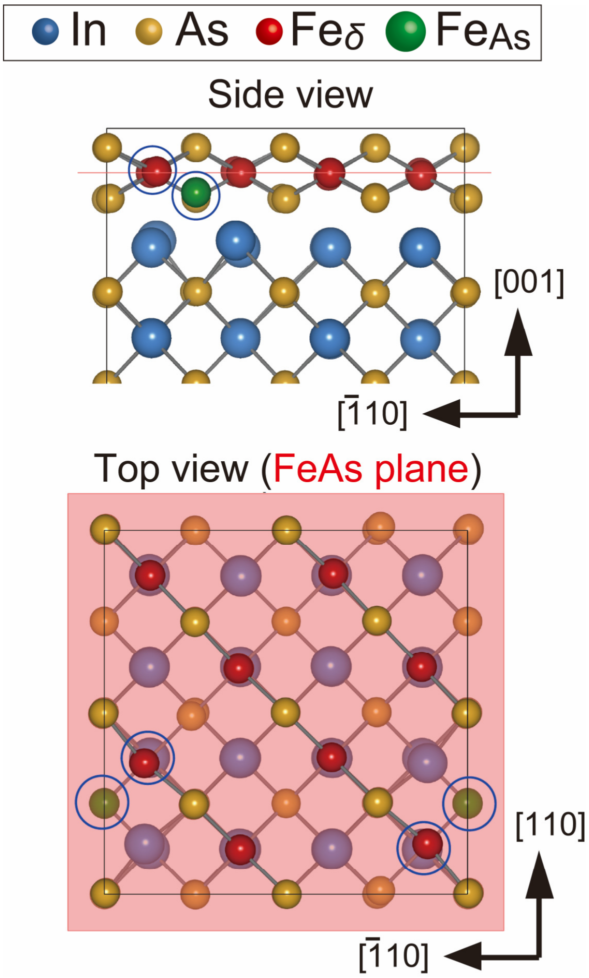

Supplementary Fig. S2| Side view and top views of atomic positions in the case there is one As-antisite Fe (Fe_{As}, green ball) point defect, obtained by our first principles calculations with the VASP code. The nearest Fe_{As} and Fe_{δ} atoms attract each other, possibly due to their strong ferromagnetic coupling, resulting in the smaller atomic distance (0.1993 nm) than the Fe-As bond length (0.2295 nm).

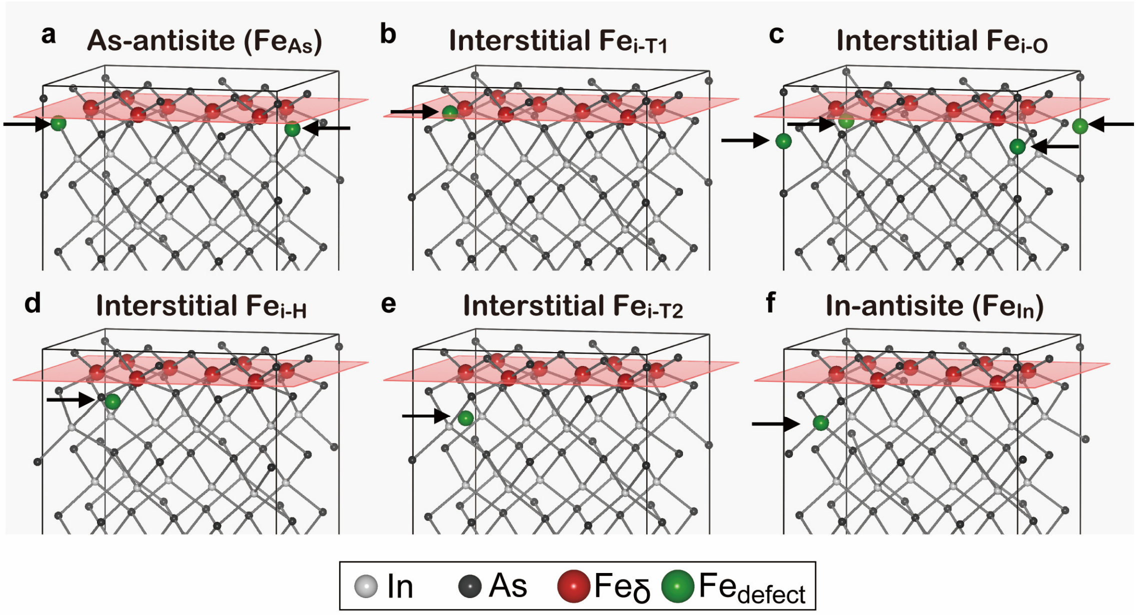

Supplementary Fig. S3| Several possible Fe-defect positions (green balls, pointed by black arrows) assumed in our first principles calculations, as also summarized in Supplementary Table S1. The substitutional Fe_δ atoms in the FeAs layer (red plane), In atoms and As atoms are shown by red, gray, and black balls, respectively.

Supplementary Table S1| Atomic distance from the nearest Fe_δ and formation enthalpy of several Fe-defect types, in the order of their distance from the substitutional Fe_δ atom in the FeAs layer, calculated by first principles calculations with the VASP code.

Position in Sup. Fig. S3	Fe-defect type	Distance from Fe_δ (Å)	Formation enthalpy (eV)
(a)	As-antisite (Fe_{As})	1.993	2.280
(b)	Tetragonal interstitial site 1 ($Fe_{i\text{-}T1}$, in the FeAs plane)	2.293	1.101
(c)	Octahedral interstitial site ($Fe_{i\text{-}O}$)	2.325	0.435
(d)	Hexagonal interstitial site ($Fe_{i\text{-}H}$)	2.379	0.443
(e)	Tetragonal interstitial site 2 ($Fe_{i\text{-}T2}$, in the next InAs plane)	2.860	-0.311
(f)	In-antisite (Fe_{In})	3.665	1.893

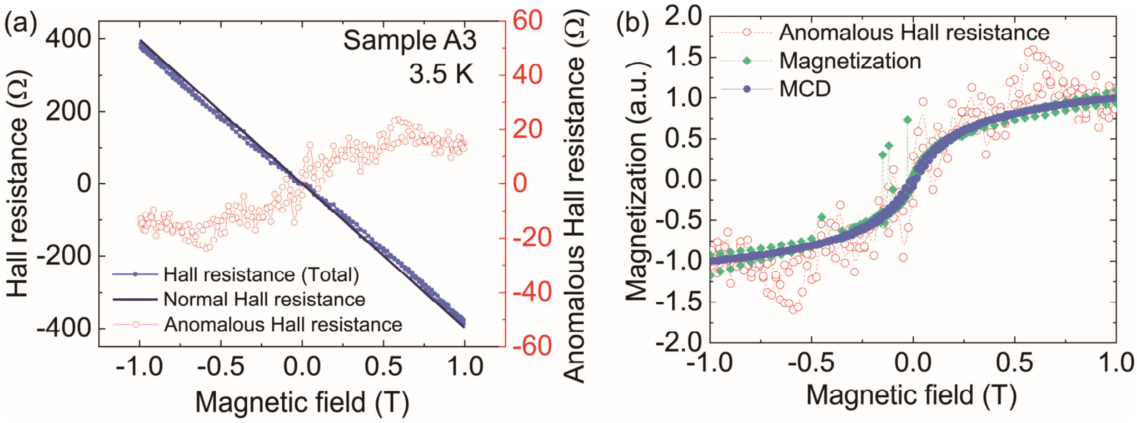

Supplementary Fig S4| Anomalous Hall resistance in the FeAs/InAs superlattices. (a) Hall resistance (purple circles), which includes a normal Hall resistance (dark blue line) and an anomalous Hall resistance (red open circles, right axis), measured in sample A3 at 3.5 K. (b) Normalized hysteresis loops measured with Hall measurement, MCD, and SQUID in sample A3.

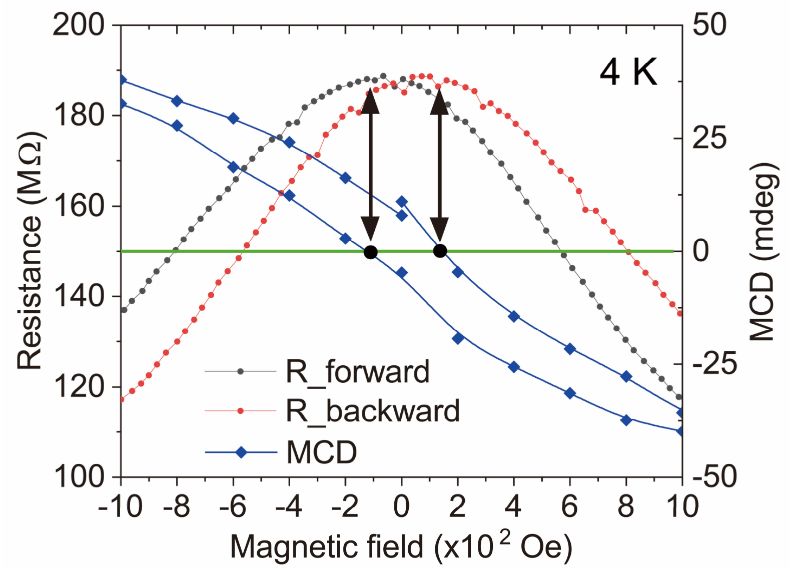

Supplementary Fig S5| Comparison between the coercive forces measured with MCD (blue diamonds) and with magnetoresistance (black and red circles) at 4 K in sample A4. Both measurements show good agreement.

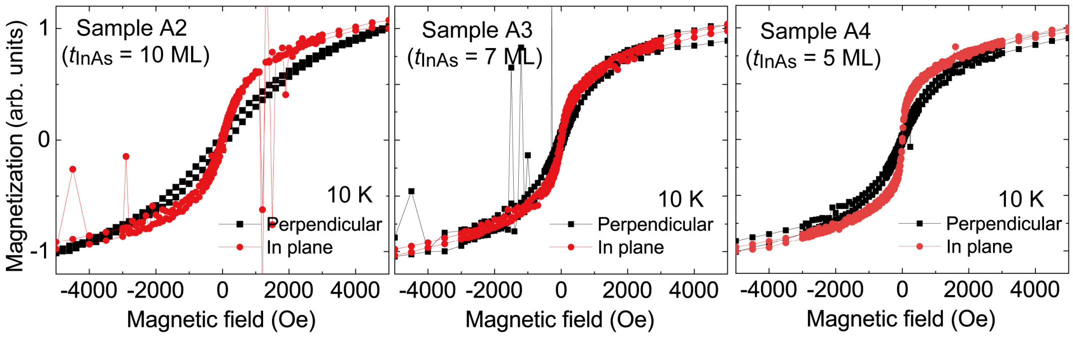

Supplementary Fig S6| Magnetization easy axis in the FeAs/InAs superlattices. (a),(b),(c) Normalized magnetization curves measured with applying a magnetic field perpendicular to (black squares) and in the plane (red circles) in samples A2, A3, and A4, respectively. All the magnetization curves were measured at 10 K by SQUID, except the curve with a perpendicular magnetic field of sample A2 that was measured by MCD. These data indicate an in-plane magnetization easy axis in these samples.

Supplementary Note 1: Magnetic moment per Fe atom of FeAs/InAs superlattices

The observation of a large magnetic moment close to 5 μ_B/Fe in our FeAs/InAs superlattices is indeed highly surprising, considering previous values reported in other Fe-As compounds. As reported by Meyer et al. [J. Chem. Phys. 143, 104302 (2015)], the magnetic moment of an isolated Fe atom can reach 6 μ_B/Fe, consisting of a spin moment of 4 μ_B/Fe and an orbital moment of 2 μ_B/Fe. Therefore, if we consider the orbital moment contribution, a value of 5 μ_B/Fe is possible as an average value of the sum of the spin moments and orbital moments of Fe atoms in the lattice sites and defect sites of the FeAs layers, and the magnetic moments of electron carriers which are also spin-polarized due to the interactions with Fe spins.

In particular, the orbital moment of a Fe atom in a compound depends strongly on the size and geometry of the compound. For example, in bulk Fe, the orbital moment is frozen out to nearly zero. However, when the cluster size is scaled down, the orbital moment of the Fe atoms at the surface/interface is known to increase due to the lower symmetry [see discussion on the scaling laws of spin and orbital moments in Meyer et al. J. Chem. Phys. 143, 104302 (2015) and the references therein]. In our FeAs/InAs superlattices, the Fe atoms are distributed only in ~1 monolayer, neighboring to the InAs layers in both top and bottom interfaces. This two-dimensional geometry maximizes the number of Fe atoms at the interfaces, which is likely to induce a large orbital moment component in the total magnetic moment. Furthermore, in the first principles calculations we described in the main manuscript and the XAFS measurements (Fig. 6), the most likely positions of Fe in the FeAs/InAs superlattice structures are the lattice sites in the FeAs monolayer (Fe_δ), the As-antisite positions (Fe_{As}), and the octahedral interstitial defects (Fe_i). The interstitial Fe_i atoms form no bonds with surrounding atoms, thus are close to the situation of an isolated Fe. This suggests that the Fe_i atoms might have a high magnetic moment close to 6 μ_B/Fe and largely contribute to the total magnetic moment. The existence of these Fe defects is different from the other Fe-As compounds usually studied in the context of Fe-based superconductors.

Regarding the magnetic moments of electron carriers, strong magnetic circular dichroism (MCD) signals (~100 mdeg, as shown in Fig. 2 in the main manuscript) indicate a largely spin-splitting band structure of the InAs host material. Furthermore, the giant magnetoresistances (~100%, as shown in Fig. 4 of the main manuscript) observed in these structures clearly indicate a strongly unbalanced spin density of states. However, since the electron density in the system is only of the order of 10^{18}~10^{19} cm^{-3}, which is two orders of magnitude smaller than the average Fe density (10^{20}~10^{21} cm^{-3}), we think

that the magnetic moments contributed from electron carriers are negligible.

Supplementary Note 2: Calculated formation enthalpy values of many Fe defects in the FeAs/InAs superlattices

We calculated the formation enthalpy values of many Fe defects, shown in Supplementary Fig. S3, using the Vienna *ab initio* simulation package (VASP) code, whose details are given in the Methods. The formation enthalpy of the octahedral interstitial Fe_i is the second lowest, after the tetragonal interstitial Fe in the next InAs layer (see Supplementary Table S1). However, we would like to note that first-principles calculations are basically valid only in the thermal equilibrium state. In the present experiments, the FeAs/InAs superlattices were grown at very low temperature (~220°C) which is a non-equilibrium MBE growth process. Thus, the situation may be considerably different from that at the thermal equilibrium state. Therefore, the antisite Fe_{As} defects may still be formed although the calculated formation enthalpy is high. We note that at this low growth temperature (~220°C), the Fe:As and In:As flux ratios are kept very close to 1:1, which are much lower than those (~1:10 to 1:20) at an equilibrium growth process of InAs at much higher temperature (450 – 500°C). This low As flux may induce the formation of As-antisite defects in our samples.

Supplementary Note 3: Anomalous Hall effect in the FeAs/InAs superlattices

We observed the anomalous Hall effect in our samples. Supplementary Figure S4a shows Hall resistance data measured in sample A3 (5 layers of FeAs) at 3.5 K. The observation is quite challenging because the anomalous Hall resistances (AHR) in these samples are small. As can be seen in Supplementary Figure S4a, the AHR component is only 5% of the total Hall resistance and is almost hidden by the normal Hall resistance component. This feature is similar to that of n-type FMS (In,Fe)As [see LDA et al., Phys. Rev. B **92**, 161201(R) (2015)]. This small AHR is expected in n-type ferromagnetic semiconductors because of the weak spin-orbit interaction in the conduction band (weaker than that in the valence band). Furthermore, the samples with strong ferromagnetism such as A2, A3 and A4 are highly resistive, which further hinders the Hall measurements. As a result, the AHR component is noisy as can be seen in Supplementary Fig. S4. Nevertheless, the magnetic hysteresis loops obtained from AHR, MCD, and SQUID measurements agree very well with others, as shown in Supplementary Fig. S4b. Moreover, as shown in Supplementary Fig. S5, the hysteresis loops measured with magnetoresistance (MR) and with MCD in sample A4 agree with each other very

well (The coercive forces are ±188 Oe). These data indicate that the ferromagnetism in the FeAs/InAs samples is intrinsic and single-phase.

Supplementary Note 4: Magnetic circular dichroism (MCD) intensity in the FeAs/InAs superlattices of samples A2, A3, A4

The magnetization per layer in all the samples are virtually same, similar values of 2.42 – 2.49×10^{-5} emu/cm^2. Therefore, the magnetization in these samples should scale up with the number of FeAs layers. Consequently, the MCD intensity, which is proportional to the magnetization, should also increase from sample A1 to A4. Indeed, the MCD intensity of sample A2 is double that of sample A1. However, it stays constant (~100 mdeg) in sample A2, A3 and A4 (see Fig. 2 in the main manuscript). We think that this is due to a short penetration depth of the incoming light in these FeAs/InAs superlattice structures: The MCD effectively measures into a depth corresponding to only three FeAs/InAs periods. This is a possible scenario, because the narrow-gap InAs host strongly absorbs visible light (200 – 800 nm). Moreover, the superlattice structrure and the mid-gap states introduced by FeAs layers may enhance the light absorption and further limit the penetration depth.
